# Polyphenols from *Salix tetrasperma* Impair Virulence and Inhibit Quorum Sensing of *Pseudomonas aeruginosa*

**DOI:** 10.3390/molecules25061341

**Published:** 2020-03-16

**Authors:** Islam Mostafa, Hisham A. Abbas, Mohamed L. Ashour, Abdelaziz Yasri, Assem M. El-Shazly, Michael Wink, Mansour Sobeh

**Affiliations:** 1 Department of Pharmacognosy, Faculty of Pharmacy, Zagazig University, Zagazig 44519, Egypt; islam_mostafa_elbaz@yahoo.com (I.M.); assemels2002@yahoo.co.uk (A.M.E.-S.); 2 Department of Microbiology and Immunology, Faculty of Pharmacy, Zagazig University, Zagazig 44519, Egypt; hishamabbas2008@gmail.com; 3 Department of Pharmacognosy, Faculty of Pharmacy, Ain Shams University, Cairo 11566, Egypt; ashour@pharma.asu.edu.eg; 4 AgroBioSciences Research Division, Mohammed VI Polytechnic University, Lot 660–Hay MoulayRachid, Ben-Guerir 43150, Morocco; aziz.yasri@um6p.ma; 5 Institute of Pharmacy and Molecular Biotechnology, Heidelberg University, 69120 Heidelberg, Germany

**Keywords:** *Salix tetrasperma*, *Pseudomonas aeruginosa*, plant pathogen, quorum sensing, virulence inhibition, molecular modeling

## Abstract

Bacterial resistance represents one of the emerging obstacles in plants, animals, and humans that impairs treatment with antibacterial agents. Targeting of the bacterial quorum sensing system is one of the strategies to overcome this problem. Recently, research has been focused on natural and food components which can function as quorum sensing inhibitors. In this study, a methanol extract from *Salix tetrasperma* stem bark was phytochemically profiled by LC-MS analysis. This resulted in the identification of 38 secondary metabolites with (epi)catechin-(epi)catechin, epicatechin, tremulacin, salicortin, and trichocarposide as the major constituents. The extracts of both stem bark and the previously profiled flower of *S. tetrasperma* were tested for anti-quorum sensing activity in a common and widely distributed pathogen *Pseudomonas aeruginosa*. The natural products inhibited swimming and swarming motilities, as well as proteolytic and hemolytic activities in a dose-dependent manner. Molecular docking of the constituents from both extracts against the quorum sensing controlling systems Lasl/LasR, rhll/rhlR, and PQS/MvfR showed that epicatechin, (epi)catechin-(epi)catechin, *p*-hydroxy benzoyl galloyl glucose, *p*-hydroxy benzoyl protocatechuic acid glucose, and caffeoylmalic acid could be the main active components. This study supports the importance of secondary metabolites, especially polyphenols, as quorum sensing inhibitors.

## 1. Introduction

*Pseudomonas aeruginosa* is a Gram-negative bacterium, which can infect plants, animals, and humans [1,2]. In plants, *P. aeruginosa* can interact with the roots leading to plant death after their colonization via formation of biofilms [3]. In humans, *P. aeruginosa* infections are associated with diabetic foot, wound, and burn infections [4,5,6]. Although several antibacterial agents are used to treat such infections, the development of bacterial resistance is considered to be a limiting factor. Thus, alternative ways to treat bacterial infections and overcome bacterial resistance are required. The use of quorum sensing inhibitors represents a new strategy that interferes with what is called virulence factors [7,8]. These virulence factors include protease, elastase, hemolysin, and pyocyanin, as well as swimming, swarming and twitching motilities, and biofilm formation. They are all under the control of quorum sensing genes and activated when bacterial cell concentrations reach a critical point [9].

In *P. aeruginosa*, Lasl/LasR, rhll/rhlR, and PQS/MvfR represent quorum sensing systems that are induced by the autoinducers 3-oxododecanoylhomoserine lactone, butanoylhomoserine lactone, and 2-heptyl-3-hydroxy-4-quinolone, respectively [4,10,11]. These autoinducers are responsible for bacterial cell communications [12]. They are secreted by bacterial cells and bind to corresponding receptors when their secreting cells have reached a specific number. Binding induces the expression of virulence factor genes and, consequently, bacterial pathogenesis [13].

Phenolic and flavonoid compounds exhibit anti-quorum sensing activity [14,15]. The genus *Salix* (family Salicaceae) is rich in phenolics, flavonoids, tannins, and saponins [16,17,18]. The Indian willow, *Salix tetrasperma* Roxb. is native to South East Asia and India. A recent study reported substantial peripheral and central analgesic, anti-inflammatory, antipyretic activities, and alleviated hyperalgesia and allodynia pain responses associated with neuropathy. These activities were attributed to the presence of 38 secondary metabolites among them rutin, kaempferide 3-*O*-glucoside, trichocarposide, coumaroylquinic acid, and salicin [19]. Salicin is a precursor of salicylic acid, which inhibits cyclooxygenase I and II (COX I and COX II), important key enzymes in the formation of mediators for pain, fever, and inflammation [18]. A previous investigation of *S. tetrasperma* bark resulted in the identification of *β*-sitosterol, *β*-sitosterol acetate, *β*-sitosterol-*O*-glucoside, *β*-amyrin, friedelin, 3*β*-friedelinol, and palmitic acid [20].

In this work, the chemical composition of the methanol extract of *S. tetrasperma* stem bark was comprehensively characterized utilizing LC-MS/MS (Figures S1–S5). We also investigated the activity of stem bark and flower extracts as quorum sensing inhibitors using *P. aeruginosa* as a model organism. Additionally, a molecular modeling study utilized binding domains of Lasl/LasR, rhll/rhlR, and PQS/MvfR to further understand the experimental findings.

## 2. Results

### 2.1. Chemical Composition

**Figure 1:**
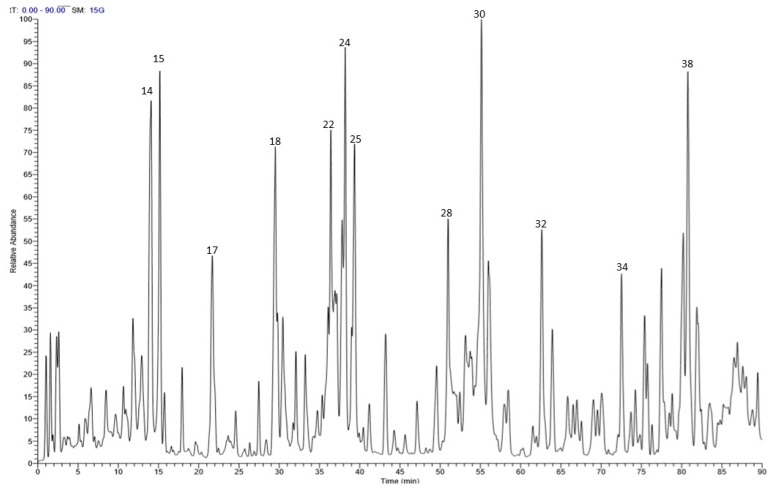
Profile of *S. tetrasperma* stem bark using LC-MS.

Liquid chromatography coupled with mass spectrometry (LC-MS) was utilized in this study to characterize the chemical composition of the stem bark extract. Altogether, 38 secondary metabolites were detected presenting the following four different categories: Phenolic acids, tannins, flavonoids, and fatty acids. (epi)Catechin-(epi)catechin, (epi)catechin, tremulacin, salicortin, and trichocarposide dominated the extract. Figure 1 illustrates the LC-MS profile of the extract and Table 1 describes the tentatively identified compounds in the extract. As for the flower extract, its chemical constituents were previously explored and documented [19]. Rutin, kaempferide 3-*O*-glucoside, trichocarposide, coumaroylquinic acid, and salicin were the main components in the flower extract among 38 secondary metabolites. A different pattern was observed between the stem bark and the flower extract: Flavonoids and phenolic acids dominated the flower extract, while proanthocyanidins and phenolic acids prevailed in the stem bark extract. In particular, a series of monomers (catechin and its isomer epicatechin and (epi)gallocatechin), dimers ((epi)catechin-(epi)-gallocatechin, (epi)catechin-(epi)catechin), and trimers ((epi)gallocatechin-(epi)catechin-(epi)catechin and (epi)catechin-(epi)catechin-(epi)catechin) were identified only in the bark extract.

**Figure 2:**
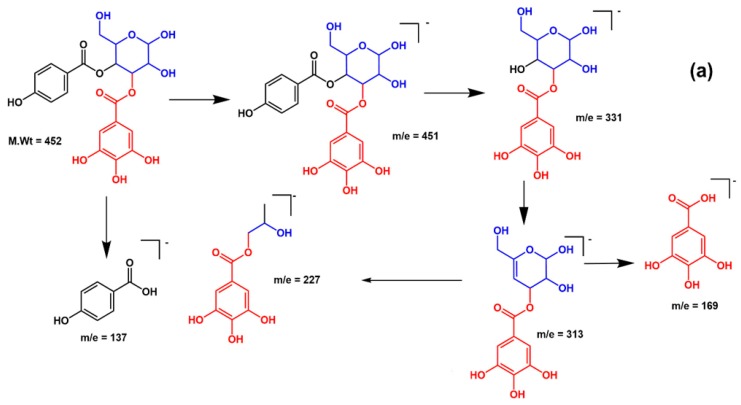

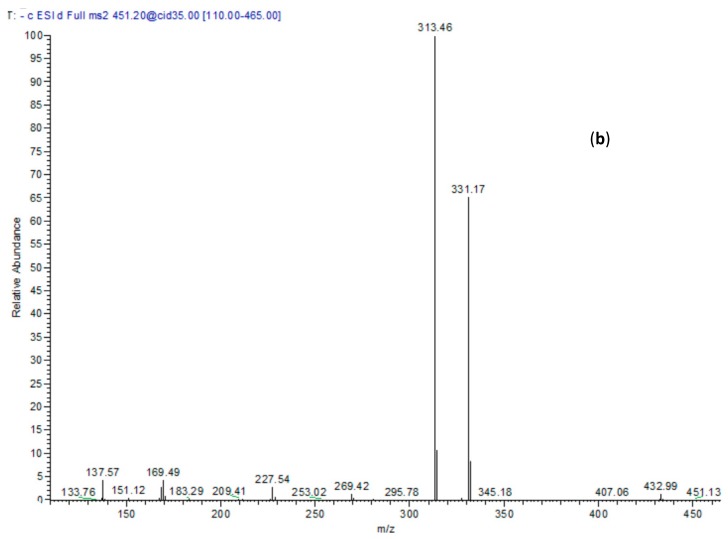
(**a**) A proposed fragmentation pattern of *p*-hydroxy benzoyl galloyl glucose at [M – H]^−^ *m*/*z* 451; (**b**) Recorded spectra (MS^2^) by ESI negative ion mode.

**Figure 3:**
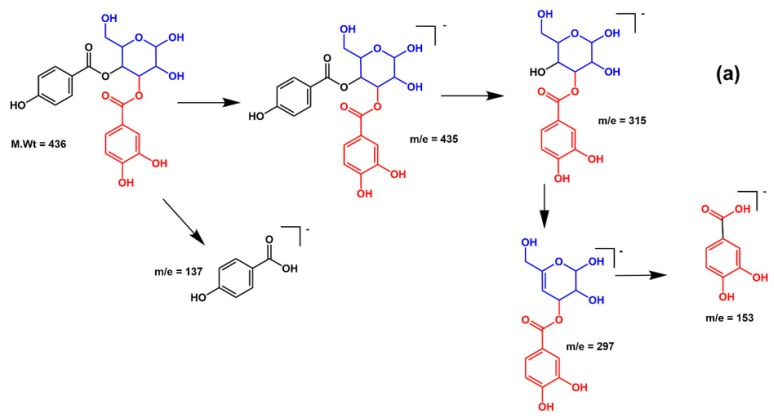

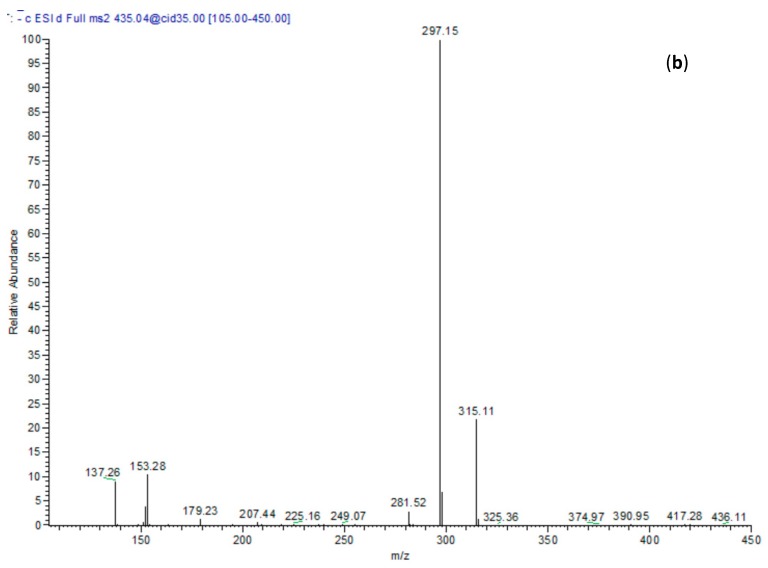
(**a**) A proposed fragmentation pattern of *p*-hydroxy benzoyl protocatechuic acid glucose at [M – H]^−^ *m*/*z* 435; (**b**) Recorded spectra (MS^2^) by ESI negative ion mode.

Compound **18**, retention time 29.58 min, exhibited a [M – H]^−^ at *m*/*z* 451 and three daughter ions at *m*/*z* 169 [M–H–120–162], 313 [M–H–120–18], 331 [M–H–120], was characterized as *p*-hydroxy benzoyl galloyl glucose (Figure 2a,b). Compound **19**, retention time 30.45 min, demonstrated a [M – H]^−^ at *m*/*z* 435 and three fragments at *m*/*z* 153 [M–H–120–162], 297 [M–H–120–18], 315 [M–H–120], was identified as *p*-hydroxy benzoyl protocatechuic acid glucose (Figure 3a,b).

### 2.2. Antibacterial Activities

*Salix tetrasperma* stem bark and flower extracts inhibited *P. aeruginosa* PAO1 growth at a concentration of 40 mg/mL. In order to evaluate their effects as quorum sensing inhibitors, doses of 10 and 5 mg/mL representing 1/4 and 1/8 MIC were used. To ensure that these concentrations had no effect on PAO1 growth, the bacterial cells were allowed to grow overnight in LB broth in the presence and absence of 1/4 and 1/8 MIC of the investigated extracts and the absorbance of suspension culture was measured at 600 nm. The statistical calculations indicated no significant difference in growth in the presence and absence of 1/4 and 1/8 MIC of the investigated extracts, indicating that any activity could be attributed to quorum sensing but not bacterial growth inhibition. 

### 2.3. Stem Bark and Flower Extracts as Biofilm Inhibitors

**Figure 4:**
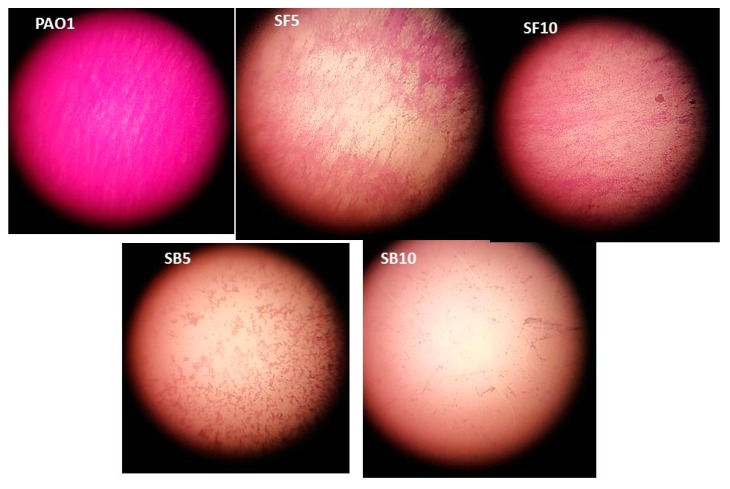
Biofilm inhibition using *S. tetrasperma* stem bark and flower extracts. PAO1, *P. aergunosa* strain; SB5, stem bark extract (5 mg/mL); SB10, stem bark extract (10 mg/mL); SF5, flower extract (5 mg/mL); SF10, flower extract (10 mg/mL). Biofilm was stained with crystal violet and visualized under light microscope (×1000).

To investigate the anti-biofilm effect, biofilm formation took place in the presence and absence of the different extracts on sterile cover slips, the formed biofilms were stained with crystal violet and examined under microscope. The treated PAO1 showed scattered cells pattern in a dose-dependent manner (lower than MIC) relative to control (Figure 4).

### 2.4. Effect on Swimming and Swarming Motilities

**Figure 5:**
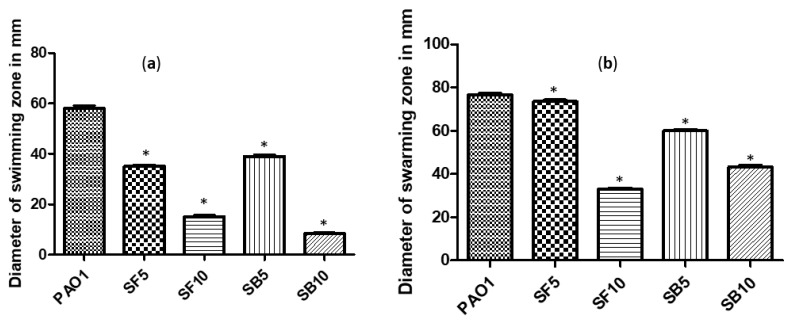
(**a**) Swimming motility inhibition by *S. tetrasperma* stem bark and flower extracts; (**b**) Swarming motility inhibition by stem bark and flower extracts. PAO1, *P. aergunosa* strain; SB5, stem bark extract (5 mg/mL); SB10, stem bark extract (10 mg/mL); SF5, flower extract (5 mg/mL); SF10, flower extract (10 mg/mL). n = 3; * significant change at * *p* < 0.05.

PAO1 motility impairment was achieved using *S. tetrasperma* stem bark and flower extracts. The extracts reduced swimming motility to 32.76% and 39.66% at a concentration of 5 mg/mL and to 85.63% and 74.14% at a concentration of 10 mg/mL (Figure 5a). The swarming motility was decreased to 21.74% and 3.91% at a 5 mg/mL concentration of the stem bark and flower extracts, and to 43.47% and 56.96% at a 10 mg/mL concentration of the same extracts (Figure 5b).

### 2.5. Inhibition of Protease Production and Hemolytic Activity

**Figure 6:**
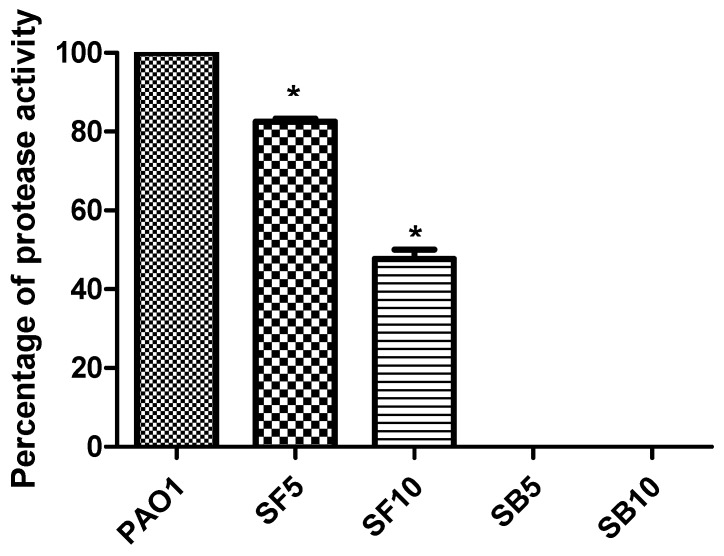
Protease production inhibition by *S. tetrasperma* stem bark and flower extracts using skim milk agar method. PAO1, *P. aergunosa* strain; SB5, stem bark extract (5 mg/mL); SB10, stem bark extract (10 mg/mL); SF5, flower extract (5 mg/mL); SF10, flower extract (10 mg/mL). n = 3; * significant change at * *p* < 0.05.

**Figure 7:**
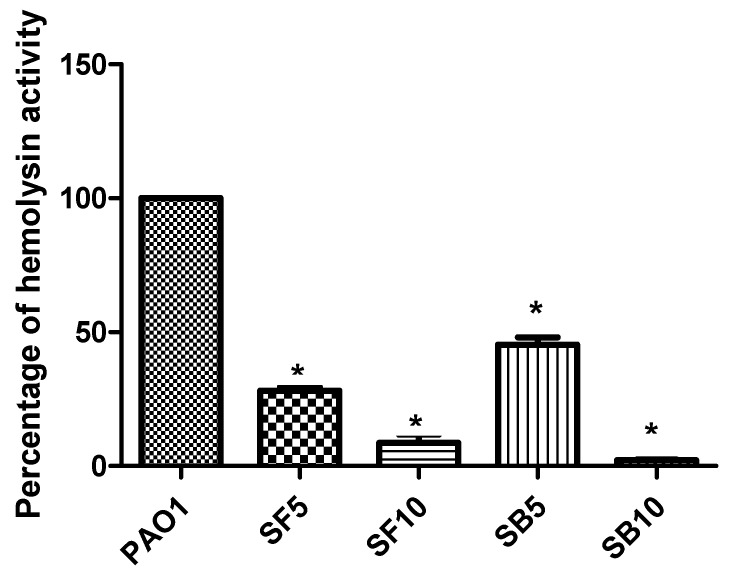
Hemolytic activity inhibition by *S. tetrasperma* stem bark and flower extracts. PAO1: *P. aergunosa* strain; SB5, stem bark extract (5 mg/mL); SB10, stem bark extract (10 mg/mL); SF5, flower extract (5 mg/mL); SF10, flower extract (10 mg/mL). n = 3; * significant change at * *p* < 0.05.

The supernatant of PAO1 cultures treated with 5 mg/mL and 10 mg/mL of the stem bark extract inhibited protease production completely at both concentrations. Treatment of PAO1 culture with the flower extract reduced protease production as indicated by reduction in proteolytic activity to values of 17.43% and 52.28% at concentrations of 5 and 10 mg/mL, respectively (Figure 6). The activity was calculated by measurement of the clearance zone around the wells containing the tested extracts in skim milk agar. The PAO1 treated cultures showed reduction in hemolytic activity by 54.65% and 71.79% as compared with untreated ones using 5 mg/mL of stem bark and flower extracts, respectively. Increasing the stem bark and flower extracts concentration to 10 mg/mL resulted in a decline of hemolytic activity to 97.76% and 91.36%, respectively. This was clear from spectrophotometric assay of the hemoglobin released from the cultures’ supernatant (Figure 7).

### 2.6. Molecular Modeling 

To gain insight about the molecular mechanism of action of the studied extracts, an in silico study was conducted using the identified secondary metabolites from the active extracts on *P. aeruginosa* LasR ligand-binding domain, RhlG/NADP active site, and PqsR active domain, which are the main systems controlling the biofilm formation. Among the docked compounds in the active site of *P. aeruginosa* LasR ligand-binding domain, only caffeoylmalic acid showed the highest fitting score within the active sites with Δ*G* equals to −41.66 kcal/mol, which is close to the enzyme ligand interaction value (Δ*G* = −59.94 kcal/mol). As seen from the amino acid interactions at the enzyme pocket, caffeoylmalic acid forms a lot of favorable interactions with the amino acid residues at the active sites evidenced by the formation of three π-bonds with ASP E:73 and ARG E:61. Additionally, two conventional hydrogen bonds are formed between the polar oxygen atoms existing in the hydroxyl and carbonyl moieties and the amino acid residues TRP E:88 and ARG E:61. In addition, van der Waals interaction exists between the carbonyl group of the compound and the amino acid residue GLY E:38 and TYR E:64; both catechin and epicatechin showed close binding energies after docking with different interaction mode; both formed a π-bond with ASP E:73 residue beside four hydrogen bonds between the phenolic OH groups and the amino acid residues ARG E:61, SER E:129, THR E:75, and LEU E:125. Other compounds failed to dock or docked but showed unfavorable interaction manifested by the positive values of the free binding energies. It is worthwhile to mention that, in the rule-based method, the phenolic groups dissociate to the negatively charged phenolate groups. These phenolate species can form a firm binding at the active sites via ionic bonding and p-bond with the amino acid residues at the active sites.

**Figure 8:**
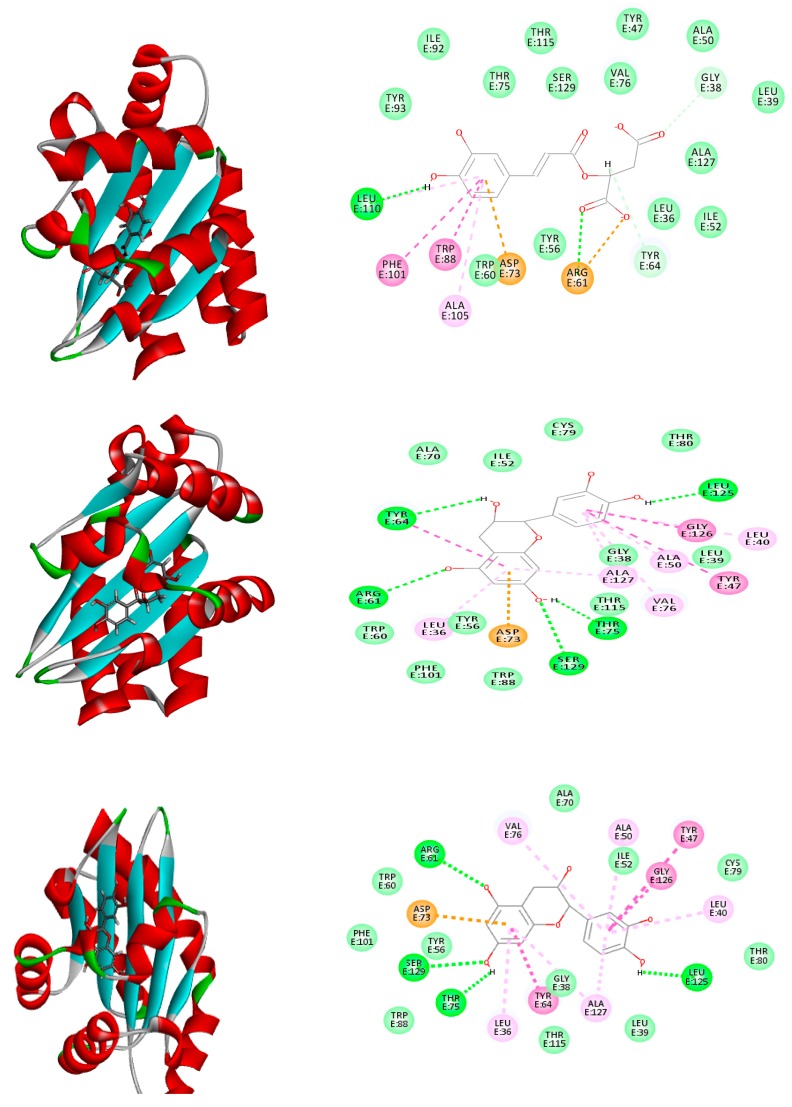
Two-dimensional (2D) (right) and three-dimensional (3D) (left) binding modes of caffeoylmalic acid (top), epicatechin (middle), and catechin (bottom) in the LasR ligand-binding domain of *P. aeruginosa* using rule-based ionization mode.

**Figure 9:**
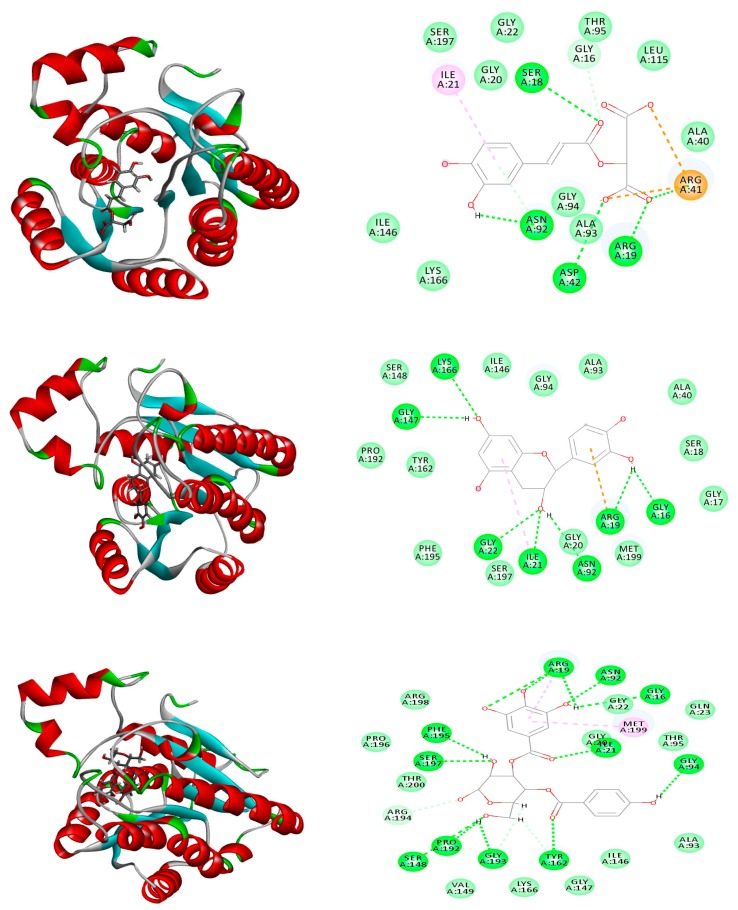
2D (right) and 3D (left) binding modes of caffeoylmalic acid (top), epicatechin (middle), and *p*-hydroxy benzoyl galloyl glucose (bottom) in the rhll/rhlR system of *P. aeruginosa* using rule-based ionization mode.

**Figure 10:**
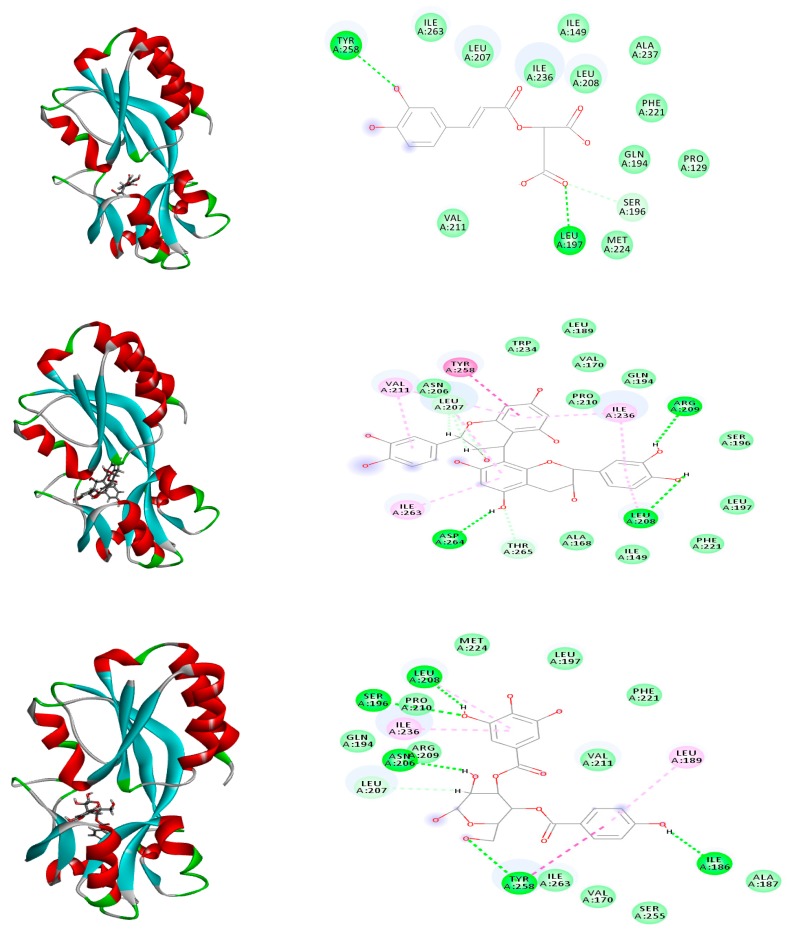
2D (right) and 3D (left) binding modes of caffeoylmalic acid (top), (epi)catechin-(epi)catechin (middle), and *p*-hydroxy benzoyl galloyl glucose (bottom) in *P. aeruginosa* quorum sensing regulator PqsR/MvfR system using rule-based ionization mode.

Similar results were noticed in the docking of compounds in the active site of rhll/rhlR system of *P. aeruginosa*. Cacffeoylmalic acid revealed the strongest affinity to the enzyme as seen in the high fitting score (−74.39 kcal/mol) that even exceeded the binding energy of the co-crystallized ligand itself (−71.47 kcal/mol) and similar binding interaction with the amino acid residues ARG A:19, ARG A:41, ASP A:42, ASN A:92, SER A:18, and GLY A:16. It is worthwhile mentioning that *p*-hydroxy benzoyl galloyl glucose and *p*-hydroxy benzoyl protocatechuic acid glucose showed binding energies (Δ*G* = −33.82, 30.41 kcal/mol, respectively), which are closely related to the values of catechin and epicatechin. The interactions of epicatechin involved the formation of hydrogen bonds with ARG A:19, ASN A:92, GLY A:16, GLY A:22, GLY A:147, ILE A:21, and LYS A:166 residues. These strong ionic bonds are attributed to the dissociation of the phenolic groups at the physiological fluid and interacting with amino residues at the active site. Other compounds showed weaker interactions as indicated by the low binding energies (Table 2 and Figure 8, Figure 9 and Figure 10).

However, the docking results on *P. aeruginosa* quorum sensing regulator PQS/MvfR system revealed a different pattern. (epi)Catechin-(epi)catechin showed the highest fitting score with binding energy equal to Δ*G* = −36.51 kcal/mol that is close to that of the co-crystalline ligand (Δ*G* = −36.14 kcal/mol). The binding involved the formation of a strong three hydrogen bonds with ARG: 209, LEU: 208, and ASP: 264 that could be explained in relation with the dissociation of the phenolic groups in the catechin and weak van der Waals bonds with THR: 265, ASN: 206, and LEU: 207. Caffeoylmalic acid, catechin. epicatechin, *p*-hydroxy benzoyl galloyl glucose, and *p*-hydroxy benzoyl protocatechuic acid glucose showed binding energies ranging from −33.62 to −26.13 kcal/mol and the binding amino acids involved were TYR:258, LEU:197, and SER:196 (Table 2 and Figure 8, Figure 9 and Figure 10).

## 3. Discussion

In this work, we comprehensively characterized the chemical constituents of *S. tetrasperma* stem bark extract using LC-MS and 38 secondary metabolites were detected. Eight compounds have been identified before from the flower extract including salicortin, trichocarposide, coumaroyl dihydrobenzoylsalicin and its isomer, tremulacin, terniflorin, hydroxy-octadecatrienoic acid, and hydroxy-octadecadienoic acid [19]. 

The increase of antibiotic resistance presents a limiting factor in the treatment of bacterial infection. Many trials have been developed to overcome this problem from which quorum sensing is a modern and valuable one. Quorum sensing inhibitors act by competition with autoinducers on their receptors. Consequently, they can inhibit biofilm formation, bacterial motility, and virulence factors [7,22]. Unfortunately, many of the previously studied quorum sensing inhibitors are clinically toxic. This challenge directed the scientists to find out alternative quorum sensing inhibitors such as FDA approved drugs and plant extracts [23,24]. Similar activities have been reported from different plant extracts, among them *Vanilla planifolia* Andrews showed anti-quorum sensing activity against *Chromobacterium violaceum* [25]. Similarly, *Conocarpus erectus*, *Bucida buceras*, *Chamaesyce hypericifolia*, *Tetrazygia bicolor*, *Quercus virginiana*, *Callistemon viminalis*, and Syzygium aromaticum extracts exhibited activity against *P. aeruginosa* quorum sensing [26,27]. Furthermore, catechin, naringenin, and taxifolin from *Combretum albiflorum* inhibited quorum sensing in *P. aeruginosa* and their activities were attributed to decreasing the expression of quorum sensing genes and production of autoinducers [15,28]. 

In this work, investigation of *S. tetrasperma* stem bark and flower extracts as a quorum sensing inhibitor was carried out. The extracts were found to inhibit PAO1 bacterial growth at a concentration of 40 mg/mL; however, concentrations equivalent to 1/4 and 1/8 MIC did not affect PAO1 viability, therefore, they were used to test the quorum sensing inhibitory effect of stem bark and flower extracts against PAO1. Quorum sensing plays a key role in biofilm formation [29]. The extracts were found to inhibit these kinds of virulence factors at doses lower than MIC. Previous studies of *Hibiscus sabdariffa* and *Moringa oleifera* extracts reflected their inhibitory effect against biofilm formation in *P. aeruginosa*, the latter exhibited similar activity in *Staphylococcus aureus* and *Candida albicans* [6,30]. 

One of the factors that affect biofilm formation is the ability of bacteria to adhere to surfaces which is linked to bacterial motility that is under the control of LasR and rhlR [31]. By targeting those enzymes; the motility is impaired, resulting in formation of weak and dispersible biofilms [32]. In this work, motility impairment was achieved using *S. tetrasperma* stem bark and flower extracts. In addition, *P. aeruginosa* can invade host tissues by the aid of hydrolytic enzymes as protease and hemolysin [33]. The inhibition of these enzymes represents a good strategy to overcome the spread of bacterial cells in the host [33]. Stem bark and flower extracts inhibited protease and hemolysin activity, and therefore they help in decreasing the spread of bacterial cells. Similar activities were reported from *Chamaemelum nobile*, *Ananas comosus*, *Musa paradiciaca*, *Manilkara zapota*, *Ocimum sanctum*, and Lagerstroemia speciosa extracts [34,35,36]. 

Molecular docking of main components of the stem bark and flower extracts revealed that their quorum sensing inhibition can be attributed mainly to epicatechin, (epi)catechin-(epi)catechin, *p*-hydroxy benzoyl galloyl glucose, and *p*-hydroxy benzoyl protocatechuic acid glucose from the stem bark extract and caffeoylmalic acid from the flower extract. Previous docking of epicatechin and proanthocyanidin from cranberry on Lasl/LasR system is in agreement with our results [37]. Another study showed that caffeoyl derivatives were found to be the main components of the anti-quorum sensing Burdock root extract [38].

## 4. Materials and Methods

### 4.1. Plant Material and Extraction

The stem bark and flowers (catkins) of *Salix tetrasperma* were collected during the spring season (30 April, 2018) from the province of Qalubiya, (Banha-Zagazig road, location 30°28′15″ N 31°14′50″ E), Egypt. Plant identity was verified by Dr. H. Abdel Baset, Professor of Botany, Faculty of Science, Zagazig University, Egypt and voucher specimen were deposited in the Herbarium of Pharmacognosy Department, Faculty of Pharmacy, Zagazig University, Egypt (Voucher specimen no SST-3 and SST-4). The stem bark and flowers were dried in shade and, then, ground into fine powder by an electric mill. Two hundred grams from each of the resulting powders were extracted twice with methanol at room temperature for a period of three days with occasional shaking. The obtained extracts were filtered and concentrated using rotary evaporator at 40 ˚C to yield 30 g and 44 g of stem bark and flower extracts, respectively. The concentrated extracts were defatted with hexane, frozen at −20 °C, lyophilized affording 26 and 38 g stem bark and flower extracts, respectively, and kept at −20 °C for chemical and biological investigations.

### 4.2. HPLC-MS Analysis

A ThermoFinnigan LCQ-Duo ion trap mass spectrometer (ThermoElectron Corporation, Waltham, MA, USA) with an ESI source (ThermoQuest Corporation, Austin, TX, USA) was used. A Discovery HS F5 column (15 cm × 4.6 mm ID, 5 µm particles) (Sigma-Aldrich Co, Steinheim, Germany) was utilized with the ThermoFinnigan HPLC system. The mobile phase was water and acetonitrile (ACN) (Sigma-Aldrich GmbH, Steinheim, Germany) (0.1% formic acid each). At 0 min, ACN was 5%, then increased to 30% over 60 min, and finally to 90% within the last 30 min. The flow rate was kept at 1 mL/min with a 1:1 split before the ESI source. The MS operated in the negative mode as previously reported [39].

### 4.3. Media and Chemicals

Luria-Bertani (LB) agar, LB broth and tryptone were purchased from Lab M Limited (Lancashire, UK). Mueller Hinton agar, Mueller Hinton broth, and tryptone soya agar and tryptone soya broth were obtained from Oxoid (Hampshire, UK). Other chemicals were of pharmaceutical grade. Bacterial strains of *Pseudomonas aeruginosa* PAO1 were kindly donated by the Department of Microbiology, Faculty of Pharmacy, Mansoura University, Egypt.

### 4.4. Minimum Inhibitory Concentration (MIC) Determination and Subinhibitory Concentration Effect on Bacterial Growth

The minimum inhibitory concentrations of stem bark and flower extracts were determined using the broth microdilution method (CLSI) [9]. The extracts were two-fold serially diluted in Mueller Hinton broth (320, 160, 80, 40, 20, 10, 5 mg/mL) and added in a volume of 100 µL to the wells of a microtiter plate. PAO1 suspension (100 µL) in Mueller Hinton broth with 1 × 10^6^ CFU/mL were added to each well. The PAO1-*S. tetrasperma* extract mixtures were incubated for 20 h at 37 °C, the minimum inhibitory concentration was defined as the lowest concentration without visible growth. Doses of 1/4 and 1/8 MIC were used to determine the effect of different extracts on growth of PAO1 as described with little modifications [40]. PAO1 culture in LB broth was inoculated overnight at 37 °C with and without the tested extracts. The turbidity of the treated and untreated cultures was determined by Biotek Spectrofluorimeter (Biotek, Winooski, VT, USA) at 600 nm.

### 4.5. Measurement of Biofilm Inhibition

The PAO1 cells were allowed to grow in TSB overnight at an optical density adjusted to OD600 = 1. The adjusted bacterial culture was inoculated with fresh media in 50 mL centrifuge tubes with sterilized cover slips in the presence and absence of 1/4 and 1/8 MIC of the investigated extracts. The inoculation took place for 16 h at 37 °C. After washing the cover slips with phosphate-buffered saline, the attached biofilms were stained with 1% crystal violet and microscopically examined using oil immersion lens (magnification power = 100×) [35].

### 4.6. Measurement of Swimming and Swarming Motilities

The effect of the bark and flower extracts on swimming and swarming motilities was studied as described by Rashid and Kornberg with few modifications [41,42]. With respect to the swimming assay, 1% tryptone, 0.5% sodium chloride, and 0.3% agar in the presence and absence of 1/4 and 1/8 MIC of the extracts were used to prepare swimming agar plates. PAO1 was cultured in tryptone broth overnight, and then diluted. Five micro-Liters of the diluted culture were stabbed into the center of the agar plates and kept at 37 °C for 24 h followed by measurement of the swimming zones. Concerning the swarming assay, another 5 µL from PAO1 overnight culture were added onto the surface of the center of dried LB swarming agar plates (1.5%) with and without 1/4 and 1/8 MIC of the extracts. After an overnight incubation of the prepared plates at 37 °C, diameters of the swarming zones were measured in mm. Pieces from the center and edges of LB swarming agar that contain vegetative and swarmer cells, respectively, were aseptically cut. The cells were cleaned from the agar pieces using phosphate buffered saline, then stained with safranine, and examined under the oil immersions lens. 

### 4.7. Measurement of Protease Inhibition

Skim milk agar method was followed to evaluate the inhibition of protease activity by the different extracts [43]. PAO1 cells were incubated in LB broth in the presence and absence of the tested extracts (1/4 and 1/8 MIC) overnight. The cultures were centrifuged at 10,000 rpm for 15 min to obtain supernatants from which 100 µL were added in the wells of 5% skim milk agar plates and incubated at 37 °C overnight. The proteolytic activity was determined by measurement of clear zones around the wells.

### 4.8. Measurement of Hemolysis Inhibition

The method of Rossignol et al. was followed to evaluate the anti-hemolytic activity of stem bark and flower extracts [44]. The supernatants of PAO1 cultures (treated and untreated with 1/4 and 1/8 MIC of the tested extracts) were used in this experiment. Briefly, 500 µL of these supernatants were added to 2% fresh erythrocyte saline suspension (700 µL), the mixtures were incubated at 37 °C for 2 h after which the hemoglobin was released by lysis of the erythrocytes, and then the lysed mixtures were centrifuged at 2500 rpm and 4 °C for 5 min. The activity was determined by measurement of absorbance at 540 nm relative to positive and negative controls that were prepared by incubating the erythrocytes in presence and absence of 0.1% SDS, respectively.

Percentage of hemolysis was determined using the equation: % hemolysis = [X − B/T − B] × 100, where X is samples (treated and untreated), B: is negative control, and T is positive control [9].

### 4.9. Molecular Modelling Study

Virtual screening of the identified compounds was done on *P. aeruginosa* LasR ligand-binding domain (PDB ID 2UV0, 1.8 A° [45]), RhlG/NADP active site (PDB ID 2B4Q, 2.3 A° [46]), and PqsR active domain (PDB ID 4JVD, 2.59 A° [47]) downloaded from protein data bank (www.pdb.org) representing the Lasl/LasR system, rhll/rhlR system, and PQS/MvfR system involved in the quorum sensing systems, respectively, using Discovery Studio 4.5 (Accelrys Inc., San Diego, USA). C-docker protocol was used and docking was performed in a similar manner as a previously described method adopting the rule-based ionization method to detect the possible influence of ionization of various active groups on the interaction of the compound at the active pocket of the enzyme [48,49]. The free binding energies were calculated in kcal/mol.

## 5. Conclusions

A total of 38 compounds were identified by LC-MS analysis of *Salix tetrasperma* stem bark extract. Screening of this extract together with the flower extract clarifies their importance in overcoming bacterial resistance by acting as quorum sensing inhibitors. The extracts were found to impair virulence of *Pseudomonas aeruginosa* by reducing its swimming and swarming motilities and decreasing its proteolytic and hemolytic activities. The results were confirmed through a molecular docking study of the extracts’ constituents against Lasl/LasR, rhll/rhlR, and PQS/MvfR systems that control the bacterial virulence.

## Figures and Tables

**Table 1:** Secondary metabolites from *S. tetrasperma* stem bark.

No.	RT	M-H *m*/*z*	MS/MS	Tentatively Identified Compounds
1	0.95	507	169, 345, 417	Gallic acid glucuronide-glucoside
2	1.05	133	-	Malic acid
3	1.37	191	111, 173	Quinic acid
4	2.29	315	153	Protocatechuic acid 3-*O*-hexoside *
5	2.60	331	125, 169, 313	Gallic acid glucoside
6	2.79	447	163, 315	Coumaric acid galloyl pentoside
7	3.46	609	305, 441, 457	(epi)Gallocatechin digallate
8	5.24	593	289, 425, 575	(epi)Catechin-(epi)-gallocatechin
9	5.91	865	289, 451, 595	(epi)Catechin-(epi)catechin-(epi)catechin
10	6.55	609	305, 423, 441	(epi)Gallocatechin-(epi)-gallocatechin
11	8.46	305	179, 221, 287	(epi)Gallocatechin
12	11.82	881	289, 577, 695	(epi)Gallocatechin-(epi)catechin-(epi)catechin
13	12.81	577	289, 407, 451	(epi)Catechin-(epi)catechin ^#^
14	13.56	577	289, 407, 451	(epi)Catechin-(epi)catechin ^#^
15	14.16	289	179, 285, 245	Epicatechin
16	15.12	289	179, 285, 245	Catechin ^#^
17	21.73	423	161, 285	Salicortin *
18	29.58	451	169, 313, 331	*p*-Hydroxy benzoyl galloyl glucose
19	30.45	435	153, 297, 315	p-Hydroxy benzoyl protocatechuic acid glucose
20	32.07	423	145, 163, 307	Grandidentatin ^#^
21	33.26	423	145, 163, 307	Grandidentatin isomer
22	36.41	477	151, 179, 315	Isorhamnetin-3-*O*-glucoside
23	37.82	431	145, 163, 307	Trichocarposide *
24	38.19	431	145, 163, 307	Trichocarposide isomer
25	39.33	431	145, 163, 307	Trichocarposide isomer
26	43.23	435	179, 273	Phlorizin
27	49.29	569	307, 423, 431	Coumaroyl dihydrobenzoylsalicin *
28	5.92	631	191, 329, 353	Chlorogenic acid derivative
29	53.02	569	307, 423, 431	Coumaroyl dihydrobenzoylsalicin isomer *
30	55.21	527	155, 405	Tremulacin *
31	56.32	577	269	Terniflorin *
32	62.93	309	171, 251, 291	Fatty acid derivative
33	70.32	311	293, 311	Eicosanoic acid
34	72.48	723	269, 453, 559, 577	Coumaroyl-terniflorin
35	75.53	271	209, 253, 271	Unidentified
36	77.26	293	171, 235, 275, 293	Hydroxy-octadecatrienoic acid *
37	79.92	295	171, 277, 295	Hydroxy-octadecadienoic acid *
38	81.01	295	171, 277, 295	Hydroxy-octadecadienoic acid isomer
39	82.39	293	113, 249, 293	Hydroxy-octadecatrienoic acid isomer

* previously described in the flower extract [19] and ^#^ previously described in *Salix subserata* [21].

**Table 2:** Free binding energies (kcal/mol) and the amino acid interaction of the identified compounds from *S. tetrasperma* stem bark and flower extracts in the active sites of Lasl/LasR system, rhll/rhlR system, and PQS/MvfR system using virtual screening program.

Compound Name	2UV0 Protein		2B4Q Protein		4JVD Protein	
	Free Binding Energy	Amino Acid Interactions	Free Binding Energy	Amino Acid Interactions	Free Binding Energy	Amino Acid Interactions
Caffeoylmalic acid	−41.66	ASP E:73, ARG E:61, LEU E:110, TRP E:88, ALA E:105	−74.39	ARG A:41, ARG A:19, ASP A:42, ASN A:92, SER A:18, GLY A:16	−30.09	TYR:258, LEU: 197, SER:196
Rutin	FD	--	−1.51	ARG A:194, ARG A:19, ASP A:42, ASN A: 92, SER A:197, MET A:199, TYR A:162, PHE A:195, GLY A:94, GLY A:20; THR A A:95	Unfavorable binding	--
Quercetin-3-*O*-glucoside	Unfavorable binding	--	−4.86	GLY A:94, ASN A:92 ARG A:198, GLY A:16, ALA A:93, SER A:197	−7.04	ARG A: 209
Kaempferol-3-*O*-galactoside	Unfavorable binding	--	−1.82	GLY A:94, ASN A:92, ARG A:19, MET A:199, ALA A:93, SER A:18	−4.19	ARG A: 209, ASP A: 264, GLN A: 194, PRO A: 210
Kaempferide-3-*O*-glucoside	Unfavorable binding	--	Unfavorable binding	--	−3.46	TYR A: 258, LEU A: 197, LEU A: 208, GLN A: 194 SER A: 196, ARG A: 209
Isorhamnetin-3-*O*-rhamnoside	Unfavorable binding	--	Unfavorable binding	--	−3.41	TYR A: 258, ASP A: 264, GLU A: 259, ASN A: 206, THR A: 265
(epi)Catechin-(epi)catechin	FD	--	FD	--	−36.51	ARG A: 209, LEU A: 208, ASP A: 264, THR A: 265, ASN A: 206, LEU A: 207
Epicatechin	−39.23	ASP E:73, SER E:129, THR E:75, LEU E:125	−37.91	ARG A:19, GLY A:16, GLY A:22, GLY A:147, ASN A:92, ILE A:21, LYS A:166	−26.83	ILE A: 236, ASP A: 264, LEU A: 207
Catechin	−36.66	ARG E:61, SER E: 129, THR E:75, LEU E:125	−35.51	ARG A:19, GLY A:94, ASN A:92, GLY A:147, GLY A:193, SER A:148	−26.13	TYR A: 258, LEU A: 208 LEU A: 207
Tremulacin	FD	--	−12.67	ARG A:19, ASN A:92, MET A:199, PHE A:195, SER A:197, GLY A:20, TYR A:162	−6.83	TYR A: 258, LEU A: 207, LEU A: 208, SER A: 196, LEU A: 207
Salicortin	Unfavorable binding	--	−6.31	ARG A:19, ARG A:41, ASN A:92, MET A:199, TYR A:162, GLY A:147, GLY A:16, GLY A:94	Unfavorable binding	--
Trichocarposide	Unfavorable binding	--	−15.87	ARG A:19, ARG A:41, ASN A:92, MET A:199, TYR A:162, GLY A:147, SER A:197	−12.63	ILE A:186, LEU A: 207
*p*-Hydroxy benzoyl galloyl glucose	Unfavorable binding	--	−33.82	ARG A:19, GLY A:16, GLY A:22, SER A:197, ILE A:21, TYR A:162, GLY A:94, PHE A:195, ASN A:92	−33.62	ILE A: 186, TYR A: 258, ASN A: 206, LEU A: 208, SER A: 196, LEU A: 207
*p*-Hydroxy benzoyl protocatechuic acid glucose	Unfavorable binding	--	−30.41	ARG A:19, GLY A:16, GLY A:22, GLY A:147, ILE A:21, LYS A:164, GLY A:94,	−28.96	ILE A: 186, TYR A: 258, GLN A: 151, LEU A: 207

FD, fail to dock.

## Supplementary Materials

LC-MS/MS spectra of some selected compounds from the bark extract are available online.
