# Supplementary material for: Polyphenols from Salix tetrasperma Impair Virulence and Inhibit Quorum Sensing of Pseudomonas aeruginosa
Source: Molecules. 2020 Mar 16;25(6):1341. doi: 10.3390/molecules25061341 (PMC7146421; doi:10.3390/molecules25061341)
Supplement: Supplementary file 1 [file molecules-25-01341-s001.pdf]

Supplementary

Polyphenols from *Salix tetrasperma* impair virulence and inhibit quorum sensing of *Pseudomonas aeruginosa*

Islam Mostafa [1], Hisham A. Abbas [2], Mohamed L. Ashour [3], Aziz Yasri [4], Assem M. El-Shazly [1], Michael Wink [5,*] and Mansour Sobeh [4,5,*]

[1] Department of Pharmacognosy, Faculty of Pharmacy, Zagazig University, Zagazig, 44519, Egypt
[2] Department of Microbiology and Immunology, Faculty of Pharmacy, Zagazig University, Zagazig, 44519, Egypt
[3] Department of Pharmacognosy, Faculty of Pharmacy, Ain Shams University, Cairo, 11566, Egypt
[4] AgroBioSciences Research Division, Mohammed VI Polytechnic University, Lot 660–Hay MoulayRachid, Ben-Guerir, 43150, Morocco
[5] Institute of Pharmacy and Molecular Biotechnology, Heidelberg University, Heidelberg, 69120, Germany
* Correspondence: wink@uni-heidelberg.de, mansour.sobeh@um6p.ma

LC-MS/MS spectra of some selected compounds from the bark extract.

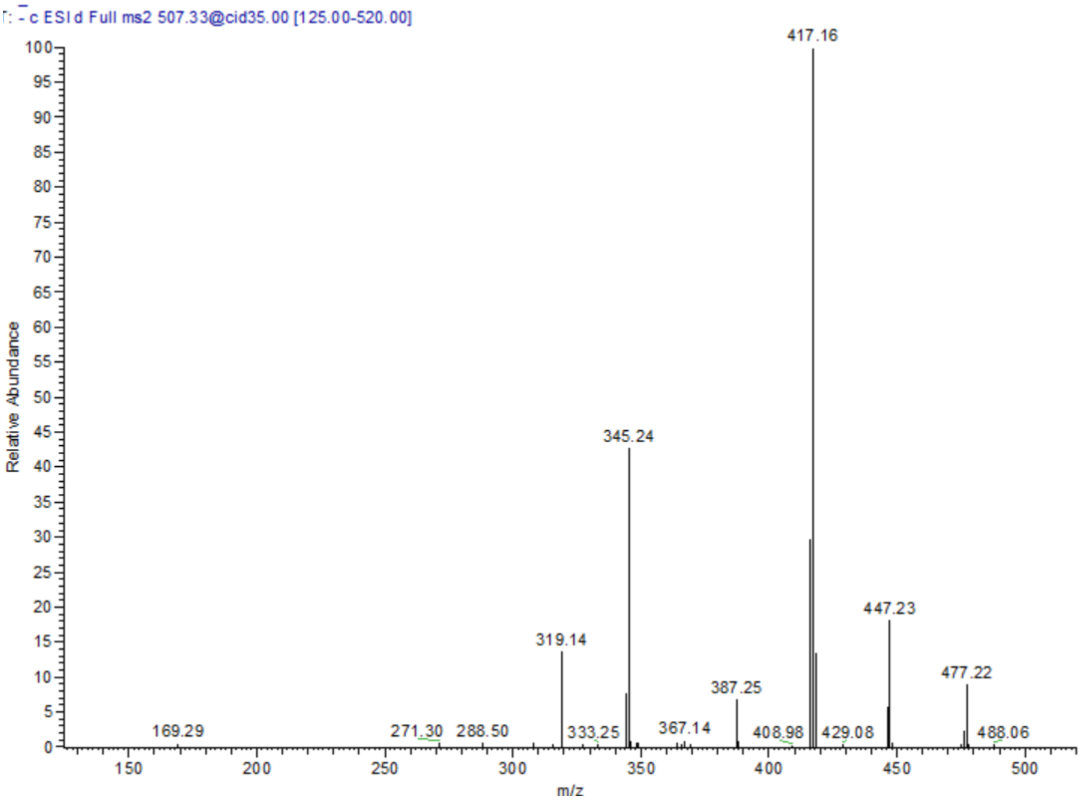

Figure S1. ESI-MS/MS of compound gallic acid glucuronide-glucoside (**1**) at [M − H]− 507

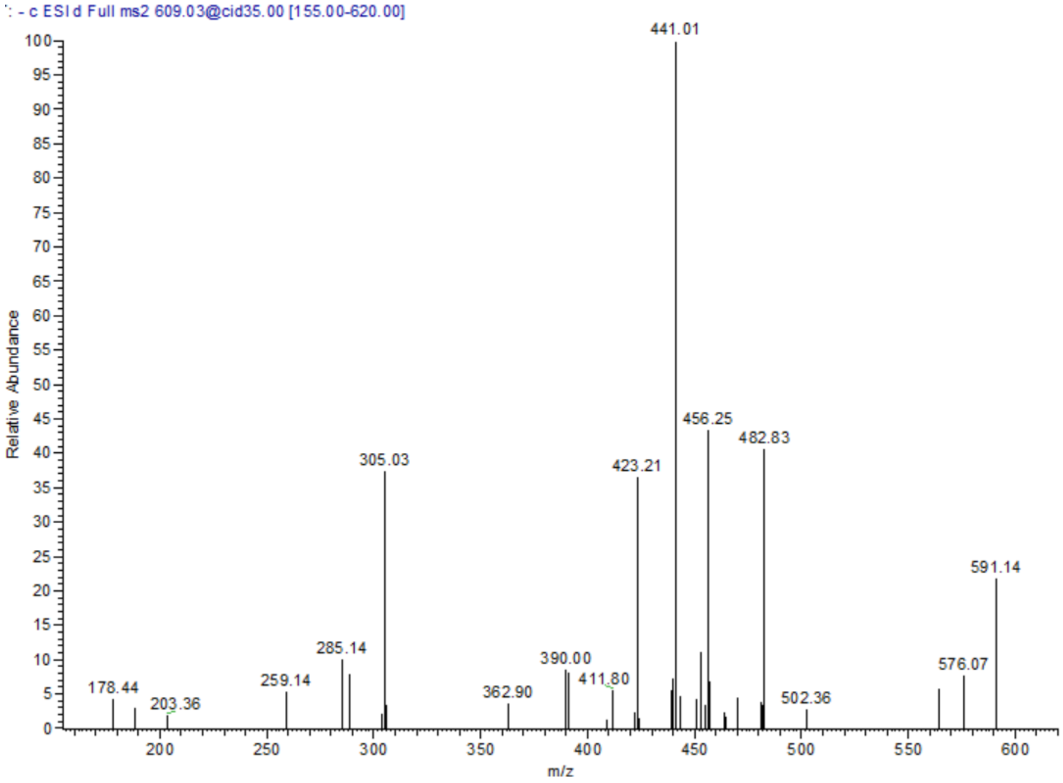

Figure S2. ESI-MS/MS of compound (epi)gallocatechin digallate (**7**) at [M − H]⁻ 609

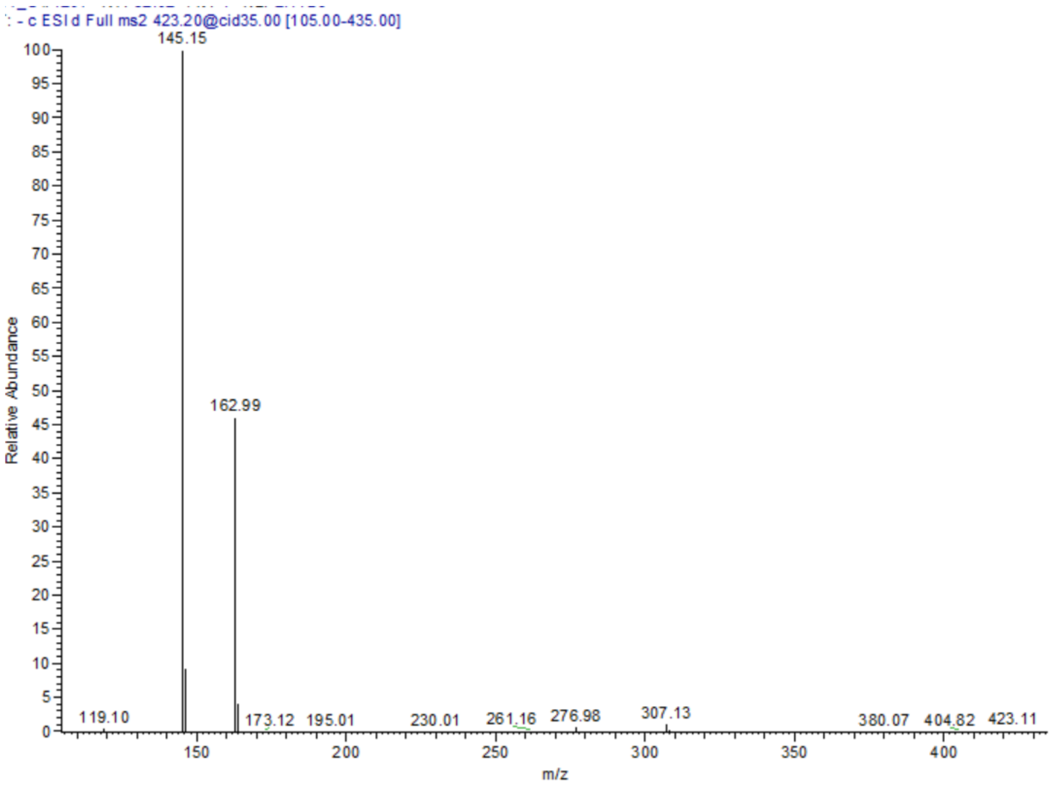

Figure S3. ESI-MS/MS of compound grandidentatin (**20**) at [M − H]⁻ 423

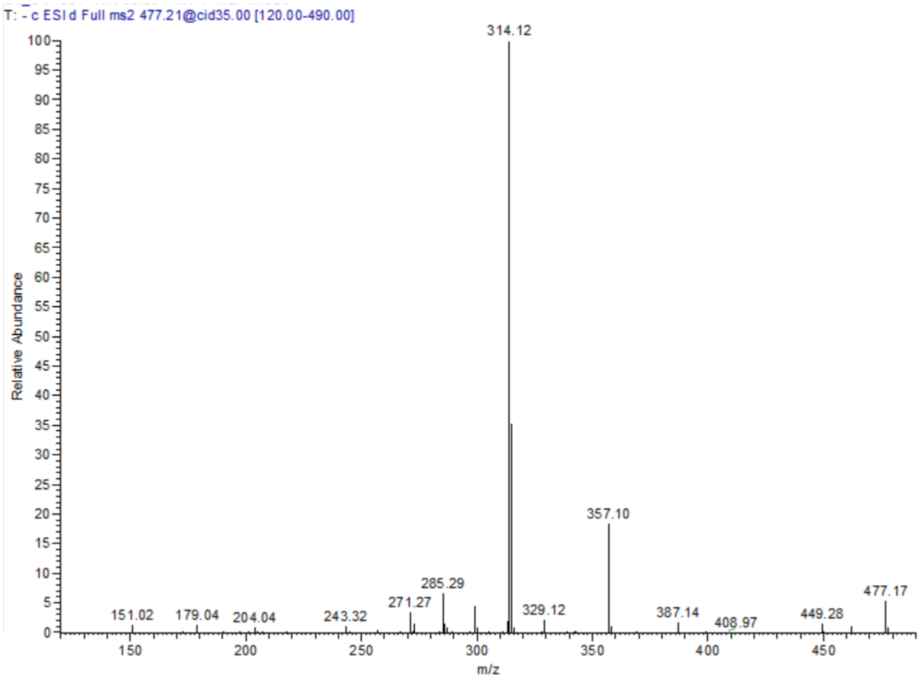

Figure S4. ESI-MS/MS of compound isorhamnetin-3-*O*-glucoside (**22**) at $[M-H]^-$ 477

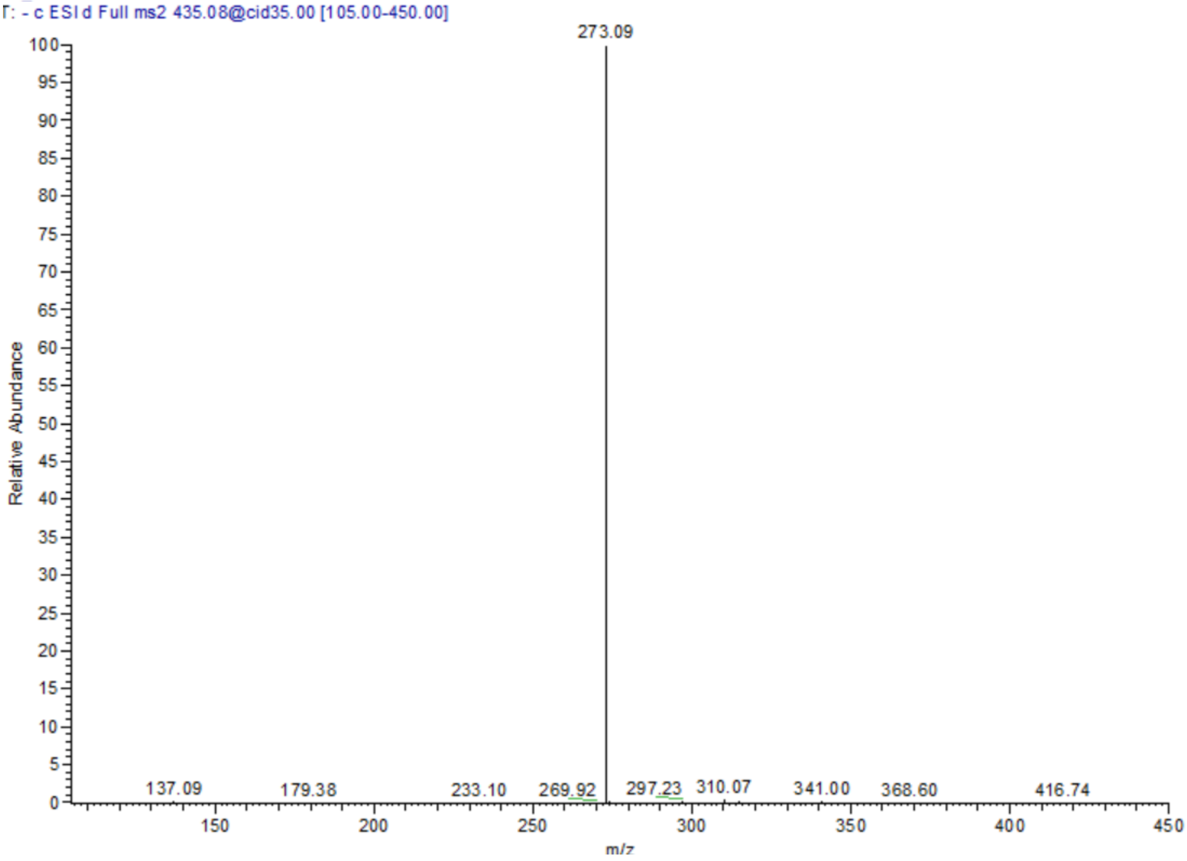

Figure S5. ESI-MS/MS of compound phlorizin (**26**) at $[M-H]^-$ 435
